# Electronic structure calculations on gallium-vacancy defects in Si_1-x_Ge_x_

**DOI:** 10.1038/s41598-025-25115-z

**Published:** 2025-11-21

**Authors:** Stavros-Richard G. Christopoulos, Emmanuel Igumbor, Edwin Mapasha, Alexander Chroneos

**Affiliations:** 1 Department of Computer Science, School of Computing and Engineering, University of Huddersfield, Huddersfield, HD4 6DJ UK; 2 Centre for Computational Science and Mathematical Modelling, Coventry University, Coventry, CV1 2TU UK; 3 Department of Mechanical Engineering Science, University of Johannesburg, Johannesburg, South Africa; 4 Department of Physics, University of Pretoria, Pretoria, 0002 South Africa; 5 National Institute for Theoretical and Computational Sciences (NITheCS), Private Bag X1, Matieland, South Africa; 6 Department of Electrical and Computer Engineering, University of Thessaly, Volos, 38221 Greece; 7 Department of Materials, Imperial College London, London, SW7 2AZ UK

**Keywords:** Si_1 − x_Ge_x_, Defects, Gallium, DFT, Doping, Binding energy, Condensed-matter physics, Nanoscale materials

## Abstract

Silicon germanium (Si_1 − x_Ge_x_) has emerged as a mainstream nanoelectronic material and as such its defect processes and energetics are technologically important. In semiconductor alloys the interaction of intrinsic point defects such as vacancies with dopant atoms are critical for the physical properties of the material and impact nanoelectronic device performance. Gallium (Ga) is a *p*-type dopant in elemental and alloys group IV semiconductors and its interaction with vacancies can impact its diffusion and electronic properties. The gallium-vacancy (Ga*V*) defect pairs are not thoroughly investigated in Si_1 − x_Ge_x_ random semiconductor alloys. Here we employ hybrid density functional theory (DFT) to study the electronic properties and binding energies in seven compositions of Si_1 − x_Ge_x_. The prediction of the prevalent Ga*V* pair in each composition is hindered by the large number of local environments that impact in turn the energetics of the defect pairs. To overcome this, we applied the special quasirandom structures (SQS) method and considered the lowest binding energy Ga*V* pairs to the favourable one for every respective composition.

## Introduction

The use of alternative to SiO2 high dielectric (high-*k*) constant materials^1–3^ has allowed the use of higher mobility semiconductor materials (as compared to silicon (Si)) for nanoelectronic applications including Si_1 − x_Ge_x_ and germanium (Ge)^4–9^.

Gallium (Ga) is a group III element and it typically acts as an acceptor atom in Si, Si_1 − x_Ge_x_ and Ge and as such it is a typical dopant alongside boron (B) and indium (In) in *p*-type regions of nanoelectronic devices^10–12^. The properties of Ga dopants in group IV elemental (i.e. Si or Ge) semiconductors have been thoroughly investigated both from an experimental and theoretical perspective^13–18^. Conversely, there is limited information for the interaction of Ga with intrinsic defects in binary group IV semiconductors (i.e. Si_1 − x_Ge_x_), particularly when considering high Ge-content solid solutions.

From a theoretical viewpoint it is not straightforward to employ DFT calculations to study even simple defect clusters in random alloys and solid solutions such as Si_1 − x_Ge_x_. This is because the energetics of the defect clusters will depend upon the nearest neighbour environments and to calculate all the possible configurations in a large supercell that will rigorously describe these local environments is practically intractable. Conversely, the SQS method^19^ allows the reproduction of the vast local environments that are present in solid solutions concurrently reducing not only the number of calculations but also the supercell size as it has been demonstrated in previous studies including binary (Si_1 − *x*_Ge_*x*_, Sn_1 − *x*_Ge_*x*_) and ternary (Si_1 − *x*−*y*_Ge_*x*_Sn_y_) group IV random alloys^20–23^. These methods can make computationally tractable the investigation of more complicated defects such as the vacancy-gallium configurations in Si_1 − x_Ge_x_.

Here we employ DFT simulations to identify the energetically favourable vacancy-gallium configurations in Si_1 − x_Ge_x_. We focus on the study of the influence of nearest neighbour environments on the vacancy-gallium binding energies and the electronic structure.

## Computational methods

We calculated the binding energies of Ga substitutional atoms with vacancies in Si_1 − x_Ge_x_ using the plane wave DFT code CASTEP^24,25^. For each of the seven compositions of Si_1 − x_Ge_x_ (x = 0.125, 0.25, 0.375, 0.5, 0.625, 0.75, 0.875) we performed 128 calculations covering all the unique distinct Ga*V* pairs, 32 calculations for all the different Ga sites, 32 calculations for all the unique different vacancy sites and one calculation for the bulk structure (i.e. a total of 1351 calculations). The vast number of DFT calculations confined the binding energy calculations to 64-atomic site supercell that were formed by two 32-atoms SQS cells the efficacy of which was discussed in previous work^26^. Correlation and exchange interactions were described by employing the corrected density functional of Perdew, Burke, and Ernzerhof (PBE)^27^, the generalized gradient approximation (GGA) was used with ultrasoft pseudopotentials^28^. For the plane wave basis the level of convergence of the atomic energies was set to 0.000544 eV/atom in conjunction with a 2 × 2 × 2 Monkhorst-Pack (MP)^29^ k-point grid. To automate the setup of calculations we used the Defects and Impurities Setup (DIMS) tool^30^, whereas the visualizations were generated using the VESTA software (version 3)^31^.

The binding energy ($\:{\text{E}}_{\text{b}}\left(\text{G}\text{a}\text{V}\right))\:$of a Ga*V* defect pair in Si_1 − x_Ge_x_ was calculated via the following relation:

1 $$\:{\text{E}}_{\text{b}}\left(\text{G}\text{a}\text{V}\right)=\text{E}[\text{G}\text{a}\text{V}{]}_{\text{s}\text{u}\text{p}\text{e}\text{r}\text{c}\text{e}\text{l}\text{l}}+\text{E}[\text{S}\text{i}\text{G}\text{e}{]}_{\text{s}\text{u}\text{p}\text{e}\text{r}\text{c}\text{e}\text{l}\text{l}}-\text{E}[\text{G}\text{a}{]}_{\text{s}\text{u}\text{p}\text{e}\text{r}\text{c}\text{e}\text{l}\text{l}}-\text{E}[\text{V}{]}_{\text{s}\text{u}\text{p}\text{e}\text{r}\text{c}\text{e}\text{l}\text{l}}\:$$

where $\:\text{E}[\text{G}\text{a}\text{V}{]}_{\text{s}\text{u}\text{p}\text{e}\text{r}\text{c}\text{e}\text{l}\text{l}}$ is the total energy of a GaV defect in the supercell of Si_1 − *x*_Ge_*x*_, $\:\text{E}[\text{S}\text{i}\text{G}\text{e}{]}_{\text{s}\text{u}\text{p}\text{e}\text{r}\text{c}\text{e}\text{l}\text{l}}$ is the total energy of the undoped supercell, $\:\text{E}[\text{G}\text{a}{]}_{\text{s}\text{u}\text{p}\text{e}\text{r}\text{c}\text{e}\text{l}\text{l}}$ is the total energy of a single Ga atom substitutionally doped in the supercell of Si_1 − *x*_Ge_*x*_ and $\:\text{E}[\text{V}{]}_{\text{s}\text{u}\text{p}\text{e}\text{r}\text{c}\text{e}\text{l}\text{l}}$ is the total energy of a supercell containing a single vacancy.

CASTEP is known to require more effort to converge hybrid simulations (k-points, cutoffs, exact exchange), which can consume computational resources for systems with large atoms. This is highly improved using the Vienna Ab initio Simulation Package (VASP)^32,33^. Due to the high computational resources required for hybrid simulations we have used VASP to simulate the partial density of states (PDOS) The projector augmented wave (PAW) method was employed as the pseudopotential approach to separate the chemically active valence electrons from the core electrons^34^. The PBE of the GGA is known to underestimate the electronic properties of most semiconductors. The Heyd, Scuseria, and Ernzerhof (HSE) hybrid functional, which combines elements of both Hartree–Fock and PBE was used as the exchange correlation^35^. A 0.25% mixing parameter and a screening parameter of 0.2 Å^−1^ was used for the HSE. The initial relaxed geometry from the previous relaxation was used for the PDOS simulation.

## Results and discussion

### Modelling silicon germanium

SQS can be described as designed small-unit-cell periodic structures, which can adequately mimic the nearest neighbour pair and multisite correlation functions of the corresponding random substitutional alloys^19,20^. In practical terms, as they are essentially atomistic models there is a distribution of local environments that would be present in the real random alloys. In the Si_1 − x_Ge_x_ lattice the Si or Ge atoms are surrounded by Si_n_Ge_4−n_ coordination shells (where *n* = 0 to 4) and these are the local environments around the Ga*V* defect. Previous studies have showed that the SQS approach is appropriate to describe local environments in Si_1 − x_Ge_x_^36–38^. This is important as it is established that the local environments will significantly influence the dopant-defect interactions in Si_1 − x_Ge_x_^38–40^.

### Ga*V* defect

**Fig. 1:**
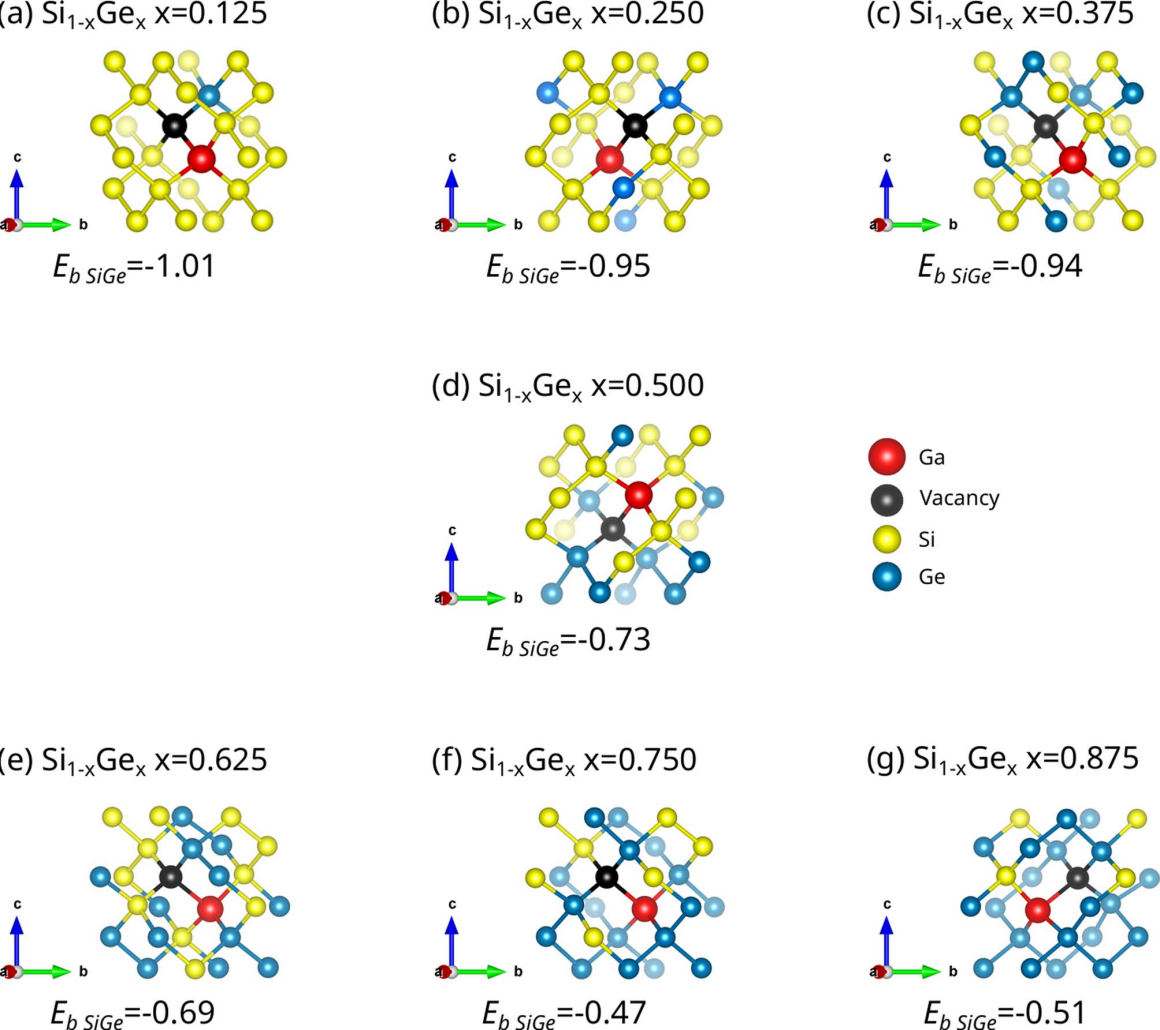
Schematic representation of the lowest binding energy (in eV) Ga*V* defects and their nearest neighbour atoms in Si_1 − *x*_Ge_*x*_ (x = 0.125, 0.25, 0.375, 0.5, 0.625, 0.75, 0.875) with the respective binding energies.

The seven 32-atom SQS Si_1 − x_Ge_x_ cells used were derived in previous work (refer to Fig. 1 in Ref^26^. Here we formed 64-atomic site supercells by using two 32-atom cells. Figure 1 represents the lowest binding energy Ga*V* defects and their nearest neighbour atoms in the different Si_1 − *x*_Ge_*x*_ alloys considered here. For all the compositions considered here the Ga*V* pair gained energy by at least one Ge atom being at a nearest neighbour position to the vacancy (refer to Fig. 1). This is a common feature in dopant-vacancy pairs in Si_1 − *x*_Ge_*x*_ as it was previously demonstrated for the *E*-centres and N*V* defect pairs^36,38^.

**Fig. 2:**
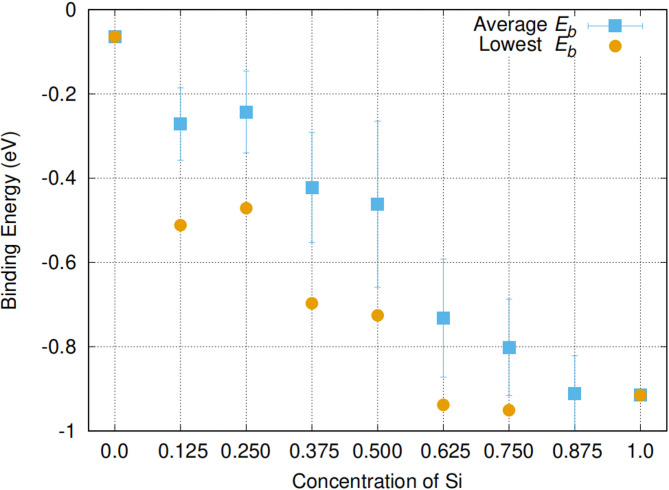
The calculated lowest and average binding energies of Ga*V* defects with respect to the Si concentration in Si_1 − *x*_Ge_*x*_ (x = 0.125, 0.25, 0.375, 0.5, 0.625, 0.75, 0.875).

Figure 2 reports the calculated lowest and average binding energies of Ga*V* defects with respect to the Ge content in Si_1 − *x*_Ge_*x*_. From this figure it is clear that there is a deviation from linearity of the lowest energy binding energies, but average binding energies are closer to linearity. In the same figure the observed range of binding energies can also be seen providing further feedback on the importance of the local atomic environment on the energetics of Ga*V* defects. The deviation from linearity is a feature of these group IV alloys (and also III-V alloys^41^. In particular, it was previously shown that the binding energies of *E*-centres (i.e. P*V* and As*V*), N*V* pairs and the diffusion properties of species facilitated by the vacancy mechanism deviate from linearity in Si_1 − *x*_Ge_*x*_ (for example^4,6,7,36,38^ and references therein) and this is consistent with the Ga*V* binding energies considered here. Of particular importance is the experimental work by Kube et al.^7^. investigating self-diffusion in Si_1 − *x*_Ge_*x*_ as it considered systematically an extensive compositional (*x* = 0.0, 0.05, 0.25, 0.45 and 0.70) and temperature (963–1543 K) indicating that there is a deviation from linearity. The evidence from experimental and theoretical work^4,6,7,36,38^ on the subtle deviations irrespective of the defect issue considered point out to that it is due to the bulk material. In a previous study, Saltas et al.^41^. considered the validity that the deviation from linearity (Vegard’s law) in Si_1 − *x*_Ge_*x*_ is due to inherent bulk properties. In particular, Saltas et al.^42^. used the c*BΩ* thermodynamic to study the impact of temperature and composition on self-diffusion in Si_1 − *x*_Ge_*x*_. Saltas et al.^42^. concluded that the deviations from linear behaviour can be traced to the diversification of the bulk properties of Si and Ge, and such as it is an inherent property of the host material, Si_1 − *x*_Ge_*x*_.

To summarize what can be derived from Figs. 1 and 2 is (a) in the most energetically favourable configurations the vacant site has always a Ge atom at a nearest neighbour site and (b) there is a significant impact of local environments on the binding energies of the GaV defect.

**Fig. 3:**
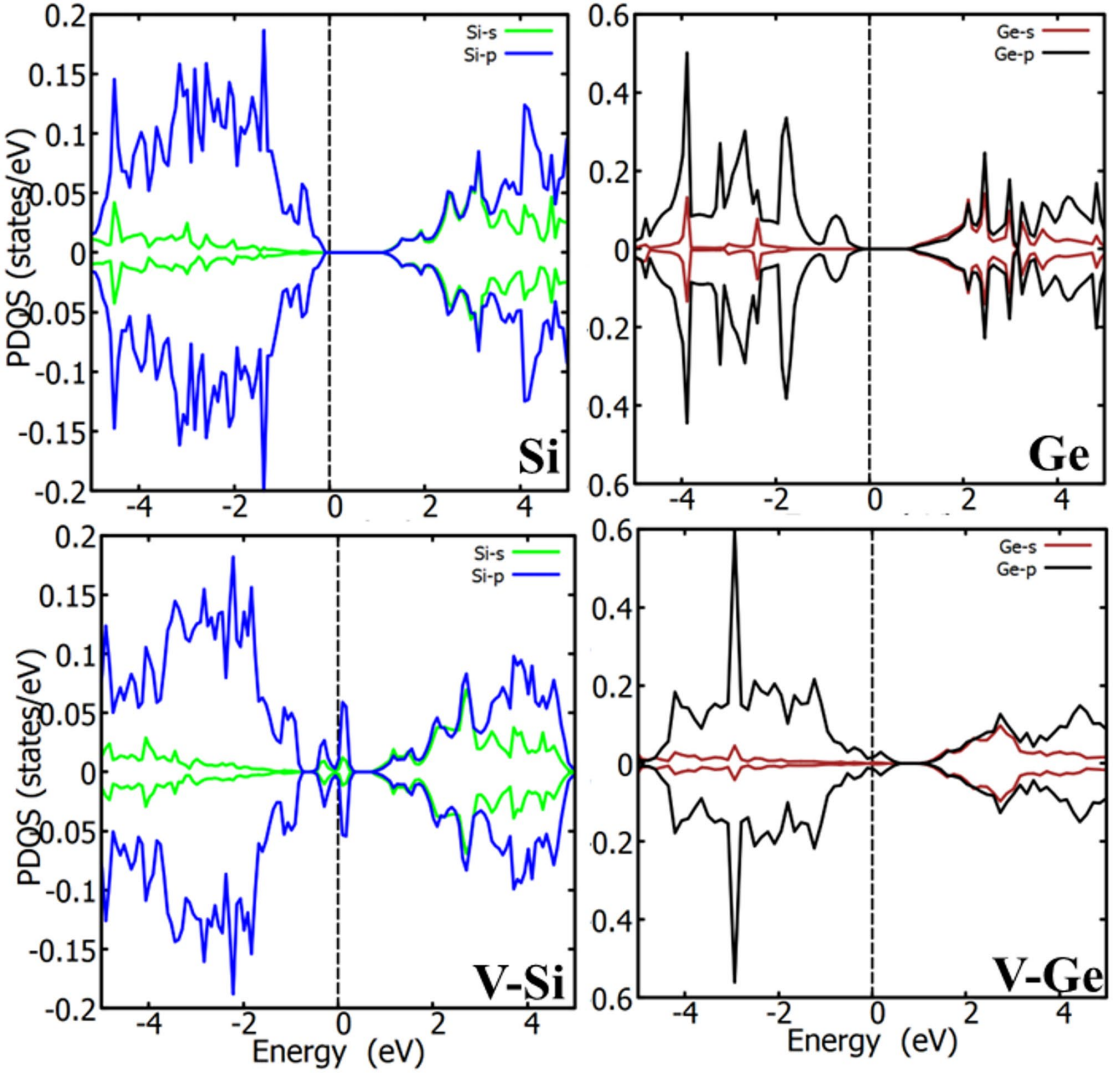
The plots of the PDOS of the pristine Si, Ge, Si vacancy (V-Si), and Ge vacancy (V-Ge). The vertical dash line is the Fermi level, set to zero.

**Fig. 4:**
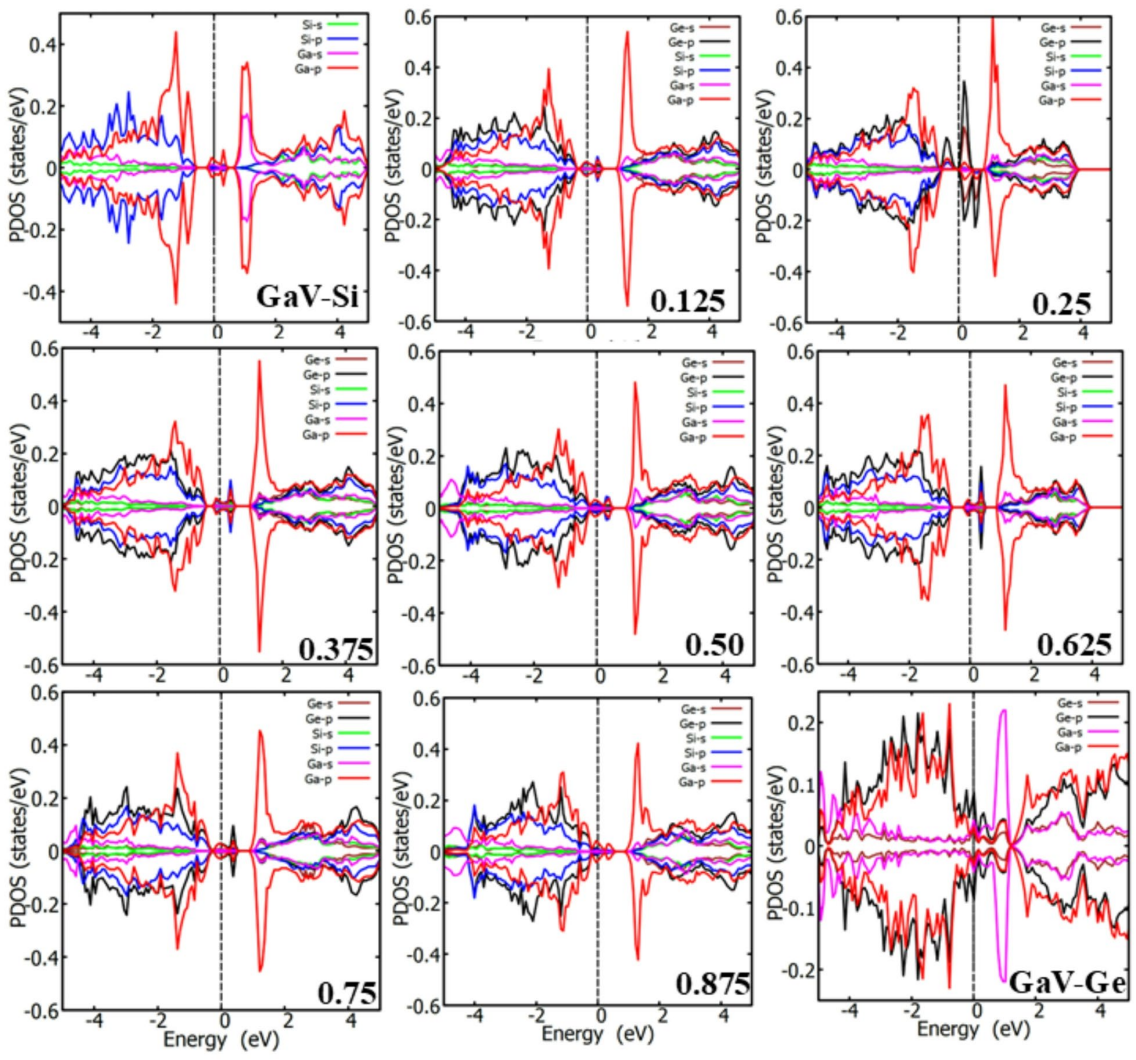
The plots of the PDOS of the GaV in silicon, GaV in Ge and Si_1 − x_Ge_x_; for x = 0.125, 0.25, 0.375, 0.5, 0.625, 0.75 and 0.875. The vertical dash line is the Fermi level, set to zero.

Figure 3 displays the partial density of states (PDOS) of the pristine Ge and Si, as well as that of the single Ge or Si vacancy in Ge or Si, respectively. Figure 4 displays the PDOS plots for Ga-doped Si_1 − *x*_Ge_*x*_ alloys at a range of concentrations (x = 0.125, 0.25, 0.375, 0.5, 0.625, 0.75, 0.875) and GaV in Si and Ge. The participating valence orbitals of the host atoms (Si and Ge) and those of the impurity atom (Ga) were plotted for the PDOS. In other words, the atoms interacting with the dopants in the presence of a vacancy were considered. However, irrespective of the Si or Ge atoms chosen, each PDOS does not significantly differ from that of the atom nearest to the dopant and vacancy defect.

The PDOS of the pristine Ge and Si suggest the absence of mid gap states. Furthermore, the Fermi level is pinned to the valence band maximum (VBM) for the Si and few eV away from the VBM for Ge. In contrast to the pristine Ge and Si, the Fermi energy of the Si_1 − *x*_Ge_*x*_ alloy shifted away from the VBM. When the concentration of Ge is low, the Fermi level of the Si_1 − *x*_Ge_*x*_ alloy mimics that of the Si-vacancy. However, when the concentration of Ge is elevated, the Fermi level of the Si_1 − *x*_Ge_*x*_ alloy mimics that of the Ge vacancy. The GaV in pure Si induced mid gap states, however, in Ge it is metallic with many ground states filling up band gap. The introduction of Ga dopant, while interacting with vacancy in the Si_1 − *x*_Ge_*x*_ alloy leads to a significant reduction in the band gap for some concentration levels. As shown in Fig. 4, numerous mid-gap states are induced within the Si_1 − *x*_Ge_*x*_ alloy due to the presence of Ga interacting with vacancy in the host system. The extent of this impact varies markedly with dopant concentration. At all doping levels, Ga dopant while interacting with vacancy introduces mid-gap states on both sides of the Fermi energy. These states are primarily contributed by the p-orbitals of the Ga atoms, in contrast to the s- and p-orbitals of the Si atom, which contribute less prominently. Furthermore, the p orbitals of Ge play a large role in the formation of these mid-gap states. The presence of mid-gap states significantly narrows the wide band gap of the pristine Si_1 − *x*_Ge_*x*_ alloy, this is in contrast to the pristine Si. In more severe cases, such as for x = 0.25 (refer to Fig. 4, the Si_0.75_Ge_0.25_ alloy is transformed into a semimetal. Strong orbital hybridizations are observed across all concentration levels, arising mainly from the p orbitals of both Ge and Ga, with additional contributions from the s orbitals of Ge. While most concentrations show negligible spin polarization, the x = 0.25 concentration exhibits pronounced spin polarization effects. This suggests that Ga interacting with vacancy doping at this level may be useful for developing spin-dependent electronic devices. However, it is worth noting that at this concentration, the Si_0.75_Ge_0.25_ alloy transitions to a metallic state. Each doping level results in different electronic behavior. Interestingly, depending on the concentration, the Ga-doped with vacancy in Si_1 − x_Ge_x_ alloy can exhibit *p*-type semiconductor characteristics, where the valence band lies above the Fermi level, as seen in Fig. 4. Across all concentrations studied, narrow peaks in the PDOS plots indicate highly localized electronic states in real space. These localized states suggest the presence of flat bands, which correspond to low group velocities and, consequently, reduced charge carrier mobility. For example, Ga dopant interacting with vacancy in Si_1 − x_Ge_x_ create highly localized states, which can influence optical transitions, affect the material’s color. Overall, the PDOS analysis of Ga-doped coupled with vacancy in Si_1 − x_Ge_x_ reveals that the defect can act as a trap for charge carriers, particularly holes, or, in more detrimental cases, degrade device performance. However, such doping can enable new functionalities, such as enhanced optical absorption, depending on the concentration.

## Conclusion

In the present study we employed systematic DFT calculations and state of the art hybrid DFT to investigate Ga*V* defects in a range of Si_1 − x_Ge_x_ compositions. It is calculated that in the most energetically favourable configurations the vacant site has always a Ge atom at a nearest neighbour site. What is also observed is that here is a significant impact of local environments on the binding energies of the GaV defect and this is reflected upon the range of the binding energies. Importantly, for all the Si_1 − x_Ge_x_ compositions considered here the Ga*V* defect pairs are bound. The Ga dopant interacting with vacancy induced mid gap states in Si_1 − x_Ge_x_, thus, significantly reduces the band gap. At x = 0.25 (refer to Fig. 1(b)), the alloy transitions into a semimetal with notable spin polarization. Depending on the doping level, the material may exhibit *p*-type behaviour or metallicity.

## Data Availability

The datasets used and/or analysed during the current study available from the corresponding author on reasonable request.
